# Positive impact of the Therasuit method on gross motor function of children with autism spectrum disorder: Case series

**DOI:** 10.3389/fneur.2023.1254867

**Published:** 2023-12-14

**Authors:** Pedro Porto Alegre Baptista, Ana Carolina Azevedo Furtado, Tiótrefis Gomes Fernandes, Renato Campos Freire Júnior, Cristiana Freitas Miranda Lima, Ayrles Silva Gonçalves Barbosa Mendonça

**Affiliations:** ^1^Laboratório de Tecnologias Assistivas e Análise do Movimento, Faculdade de Educação Física e Fisioterapia, Universidade Federal do Amazonas, Manaus, Brazil; ^2^Studio Kids Manaus, Clínica de Atendimento Infantil, Manaus, Brazil

**Keywords:** autism spectrum disorder, physical therapy, gross motor function, Therasuit, children

## Abstract

The Therasuit method is a valuable physiotherapeutic method to improve the gross motor function of children with neuromotor disorders. This series of case studies investigates the effect of the Therasuit method on the gross motor function of children with autism spectrum disorder (ASD). Therasuit method is a therapeutic intervention that involves the use of a therapeutic suit attached to a cage to stimulate gross motor skills, muscle strengthening, stretching, task training, and balance, which is a positive intervention for other neurodevelopmental disorders. The study was conducted with nine male children (42.1 + 4.1 months old) with ASD who received the Therasuit protocol for 4 weeks (20 sessions). The Gross Motor Function Measure (GMFM-88) was used to assess the children’s gross motor function before and after the Therasuit method intervention. In dimension B, several skills showed improvement, including transfer to sitting, lean forward and return, trunk rotation without support, and transfer from sitting to all four stances. In dimension C, an increase was observed in skills such as being prone to all four stance transfers and reaching above the shoulders. In dimension D, maximum scores were achieved in skills such as pulling to stand on a large bench without assistance. The dimensions with the greatest impairment were D and E, corresponding to gross motor skills in orthostasis and dynamic skills in orthostasis, respectively. The findings suggest that the Therasuit method is a promising resource for treating motor impairments in children with ASD. However, further studies with a larger sample size, an adequate control condition, and random assignment of participants would be needed to provide stronger evidence of the method’s effectiveness in this population.

## 1 Introduction

Autism spectrum disorder (ASD) is a neurodevelopmental disorder characterized by deficits in communication and social interaction, the presence of repetitive behavior patterns, restricted interests, and limitations of activities, resulting in a negative impact during the child’s development process (1). However, although central symptoms of social interaction, communication, and stereotyped behavior impairments are mostly discussed, deficits in gross motor skills or physical performance, such as gait abnormalities, postural instability, and coordination difficulties, are commonly observed in children with ASD (2, 3).

The social and behavioral impairments in children with ASD tend to bring on fewer opportunities for physical activity, negatively affecting motor behavior and possibly leading to obesity (3). Motor impairments associated with ASD show alterations in brain development processes, structures, and connectivity. Recent evidence showed an altered pattern of brain growth in individuals with ASD, particularly in the amygdala and frontal cortex (4). This abnormal pattern of frontal cortex development may be caused by an increase in the number of neurons in this area (5). Other abnormalities of the central nervous system have been reported in the cerebellum (6) and corpus callosum (7). Thus, deficits in cortical circuitry and atypical connectivity between neurological structures have been described as key factors associated with some of the prevalent symptoms, such as impairments in social functioning or repetitive and restrictive behaviors in people with ASD (8).

Motor difficulties in children with ASD are a highly relevant topic in scientific research, as they significantly influence the quality of life and development of these children. Recent studies have shown that children with ASD often exhibit motor developmental delays, alterations in muscle tone, impairments in balance and motor coordination, as well as challenges in learning and motor planning. These difficulties can drastically interfere with their lived experiences and, consequently, with the acquisition of crucial skills for participation in daily life activities, physical activities, play, and sports (9–12).

Furthermore, during early childhood (0–24 months), the learning process across various developmental domains relies on the experiences children undergo, including their ability to move and explore their surroundings. Consequently, if a child experiences motor difficulties, they are inclined to explore less and, consequently, learn less, which can lead to motor development delays in subsequent childhood stages (13).

Stereotyped movement-related behaviors, such as hand-flapping, foot-tapping, or object spinning, can also hinder the refinement of movements and skills, impacting the performance of functional tasks and affecting environmental exploration (11).

Motor interventions directed at individuals with ASD are most commonly conventional physical therapy and animal-assisted therapies, such as dog training, that help with motivation, interaction, communication, participation, and emotional regulation, with results relating to increased motor skills and a reduction of activity limitation (14, 15). Although many studies have shown physical activity and motor learning strategies being effective in children and adolescents with ASD (16–19) results are uncertain because of the scarcity of controlled protocols, high variability in intervention approaches and in time and frequency of intervention, and high variability in outcome measurement.

The Therasuit method is an intensive treatment protocol that promotes motor learning and proprioceptive improvements in patients with neuromotor disorders (20). It involves dynamic orthotics and a therapeutic suit attached to a cage for stimulating gross motor skills, muscle strengthening, stretching, task training, and balance. The treatments are customized to the child’s needs and limitations, exerting continuous tension to facilitate functional gains (21). This intervention demands a large number of diverse motor repetitions and, consequently, stimulates learning, motor control, and planning (22), making it a potential therapeutic resource for treating motor impairments in individuals with ASD.

Therasuit method could prove to be promising to children with ASD because, in this disease, it is also common to see a delay in the motor development of gross motor functions (23). The motor development delay in ASD children arises from two main sources. The first is the atypical development and functioning of the nervous system, and the second is the negative feedback loop of reduced social and environmental interaction that reduces the child’s motor demands and hinders motor development, which in turn reduces social and environmental interaction (13). The Therasuit method is a promising technique to ameliorate motor gross function in other conditions with atypical brain functioning, such as cerebral palsy (24). Furthermore, by using the Therasuit method, therapists might disrupt the negative environmental-motor feedback loop by ameliorating the capacity of gross motor function activities, which in turn increases activity performance and could improve social/environmental interaction.

Karadağ-Saygı and Giray (25) reviewed the clinical aspects of Therasuit in children with cerebral palsy and observed significant improvements in trunk stability, scapular girdle function, gross motor function, and gait. However, it is necessary to consider that the studies used the method on different age groups and functional levels (25). Nevertheless, evidence of the clinical efficacy of the Therasuit method is still scarce, and there is no proven scientific evidence of the impact of the method on children with ASD. Therefore, this study assessed the effect of the Therasuit method on the gross motor function of children with ASD.

## 2 Materials and methods

The study was approved by the Ethics Committee on Human Research of the Federal University of Amazonas, under the registration number CAAE: 55724922.9.0000.5020. Data collection, access, and publication were conducted only after parental or legal guardian approval. The parents were presented with information about the case study, and informed consent by the parents was given prior to any data collection.

A series of studies was conducted on these cases in Manaus-AM, Brazil. Nine children were recruited from a specialized pediatric physical therapy clinic. The study consisted of nine male children treated with the Therasuit protocol. The average chronological age was 42.1 months (± 4.1 months). The children did not receive any previous physiotherapy intervention, but all underwent speech therapy and were followed by a neuropsychologist and neuropediatrician. The children were patients referred to a local private practice physical therapy clinic, where the therapist identified the subject as a potential patient and was able to complete the Therasuit method protocol. Evaluations were carried out before and after the intervention using the Gross Motor Functions Measure (GMFM-88) (26) and were performed by a blind evaluator who did not participate in the intervention. Inclusion criteria were children with an ASD diagnosis showing developmental delay according to the Denver II Developmental Screening Test, obtained from the child’s medical records. Children with inconsistent diagnoses and ASD associated with other health conditions were excluded.

**Figure 1:**
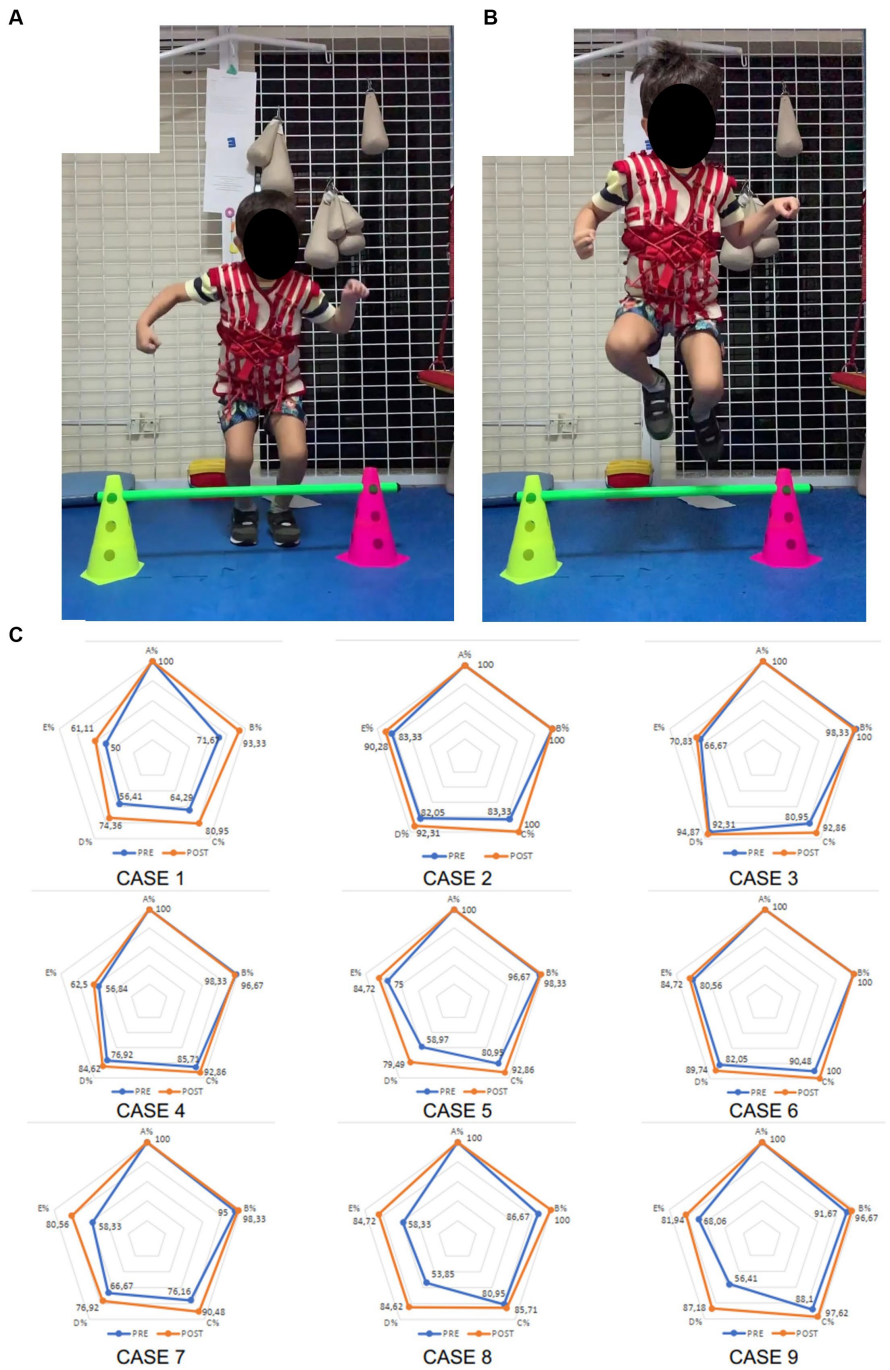
Image of the Therasuit method vest and individual cases of GMFM-88 scores. Images show the Therasuit method vest being used during the task of “jumping” in the pushing phase **(A)** and flight phase **(B)**. Image **(C)** shows the GMFM-88 dimensions percentage scores in individual radar graphs for each participant before (blue) and after (orange) intervention.

The intervention protocol consisted of the use of the Therasuit vest (dynamic orthosis) associated with kinesiotherapy. The vest of the Therasuit method is a child-sized vest fitted with elastic bands at strategic places to improve joint positioning and posture (Figures 1A,B). However, it should be associated with active (voluntary) movement performed by the subject. Preferably, while using task-oriented practice, which facilitates motor acquisition and, consequently, an increase in motor repertoire (20, 21). The method was composed of five physical therapy sessions per week for 4 weeks, for a total of 20 sessions. Each session was 3 h long and structured with the following rehabilitative components: stretching, strengthening of limb muscles, stimulation of functional postures, balance training, gait training, and stimulation of tasks while standing, sitting, in the prone position, and in the supine position. The Therasuit vest was used for at least 1 hour per session. The vest was always used at the beginning of the session and was removed when the child expressed annoyance towards the vest. Active movement during the session was elicited through verbal commands, visual clues, environment enrichment, and physical support when necessary. At times when the participant did not understand the instructions, the therapist would show the participant how to execute the task. No parental involvement was required at any point. The treating therapist was certified to use the Therasuit method and determined the exercise parameters based on the child’s limitations and impairments and specific therapeutic objectives.

The Gross Motor Function Measure (GMFM-88) was assessed before and after intervention. The GMFM-88 was designed to measure the gross motor function of children with neuromotor disorders; it has been validated for children with cerebral palsy and Down syndrome and can detect changes to it over time (26). The 88 items are grouped into five dimensions: A – lying and rolling (17 items); B – sitting (20 items); C – crawling and kneeling (14 items); D – standing (13 items); and E – walking, running, and jumping (24 items). Each item is scored from “0” to “3,” where “0” indicates no movement execution and “3” indicates complete movement. At the end of the evaluation, the total in each dimension is added and converted into a percentage about the maximum possible score in dimension (26) (Supplementary material).

The data were tested for normality using the Kolmogorov–Smirnov test. A paired *t*-test was performed to measure the effect of the intervention. The statistical significance for all tests was set at 5%. All data were processed using SPSS software (version 22).

## 3 Results

From the methodology of this study, tables and figures have been elaborated to show the results of the GMFM-88 observed before and after the Therasuit method protocol (Figure 1C).

**Figure 2:**
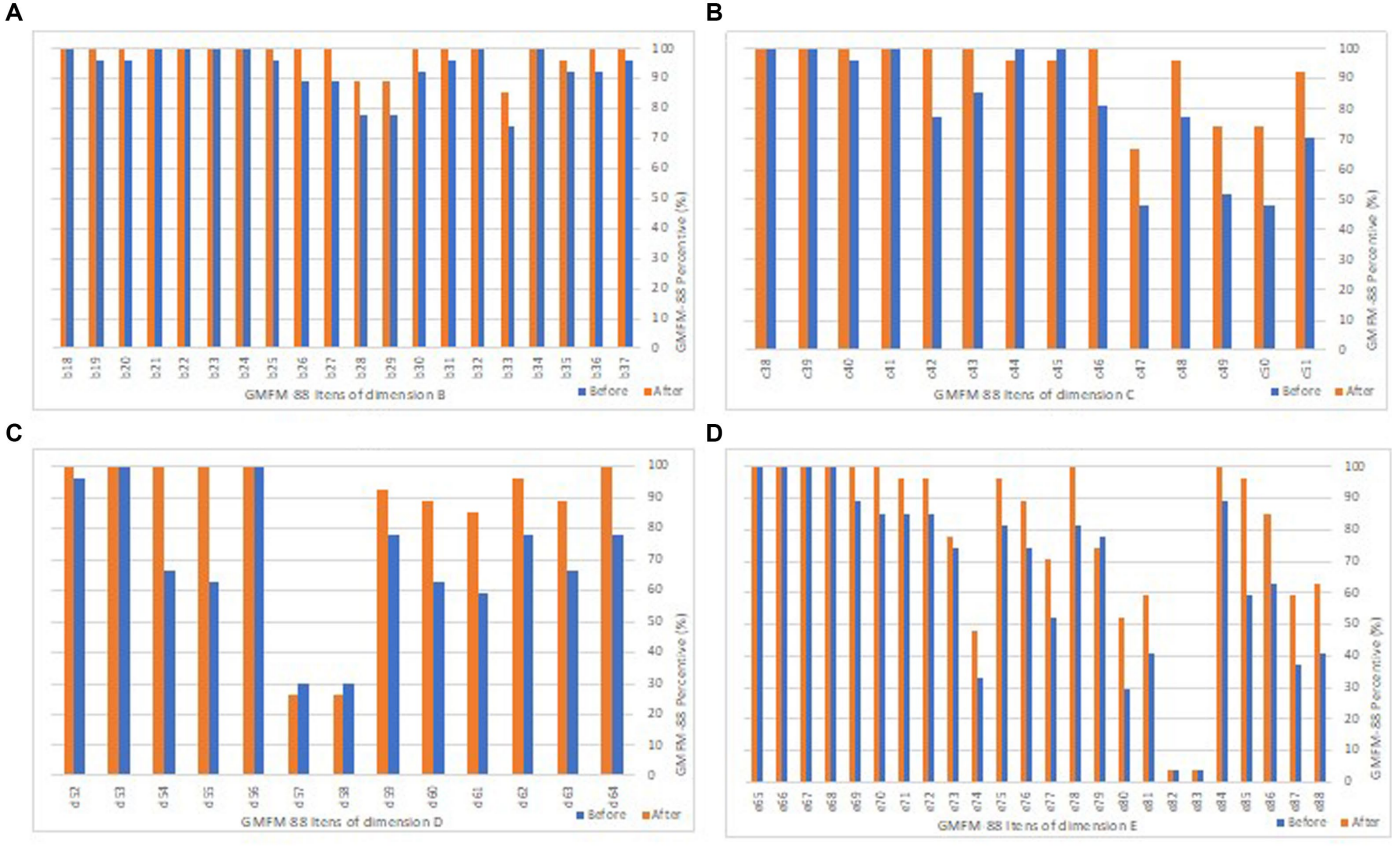
Average score of GMFM-88 items. The comparison of the score on GMFM-88 before (blue) and after (orange) the Therasuit method intervention. The X-axis is the percentage of the score. The Y-axis is the dimension (letters) and motor function (number) assessed. Dimension A is not shown. **(A)** Bar graph of the items in dimension B: sitting; **(B)** Bar graph of the items in dimension C: crawling and kneeling; **(C)** Bar graph of the items in dimension D: standing; **(D)** Bar graph of the items in dimension E: walking, running, and jumping. For further information on each motor function item, please refer to the Supplementary material.

In dimension A of GMFM-88, all children showed a maximum capacity item at both times (Table 1). Figure 2 illustrates dimensions B to E of the GMFM-88 item score before and after the intervention, and in Table 1, it is shown that the total score in GMFM-88 increased after the interventions (*M* = 8.59% ± SEM = 1.4% p.p.; *p* < 0.001).

**Table 1:** GMFM-88 before and after intervention.

	Before intervention mean (SD)	After intervention mean (SD)	Difference mean (SEM)	*p* value
TOTAL	82.1% (7.1)	90.7% (4.3)	8.59% (1.4)	<0.001*
Dimension A	100%	100%	–	–
Dimension B	93.3% (9.3)	98.0% (2.2)	4.63% (2.6)	0.12
Dimension C	81.2% (7.7)	92.6% (6.4)	11.38% (1.4)	<0.001*
Dimension D	69.5% (14.1)	84.9% (7.0)	15.39% (3.4)	0.002*
Dimension E	66.3% (11.5)	77.9% (10.5)	11.58% (2.7)	0.002*

* Statistical significance. SD, standard deviation; SEM, standard error of the mean; GMFM-88, Gross Motor Function Measure of 88 items.

In dimension B, the following skills showed improvement, reaching maximum scores: transfer to sitting (from lying) to the right (item 19); lean forward and return (item 25); perform trunk rotation without support to 45° to pick up an object to the right and left (items 26 and 27); transfer from sitting to all four stances on the right side (item 31); and sit on a large bench with feet free (item 37). In dimension C, there was an improvement after the use of the Therasuit method (*M* = 11.38% ± SEM = 1.4% p.p., *p* = 0.002) (Table 1). An increase was observed in the following skills: prone to all four-stance transfer (item 41), reaching above the shoulders in all four supports (with weight transfer to the contralateral limb) on the right and left sides (items 42 and 43), and climbing stairs on all four stances (item 46). In dimension D, the maximum score was achieved in the following skills: pull to stand on a large bench without external assistance (item 52); perform unipedal support for 3 s with unilateral self-support for the right and left foot (items 54 and 55); and pick up an object from the ground with free arms, returning to orthostasis (item 64) (Figure 2).

The dimensions with the greatest impairment were D and E, corresponding to gross motor skills in orthostasis (maintenance and transfers) and dynamic skills in orthostasis (walking, running, and jumping), respectively. Dimension D showed the most significant improvement after the Therasuit cycle (*M* = 15.39 ± SEM = 3.4% p.p., *p* = 0.002) (Table 1). Items D57 (unipedal support for 10 s on the left limb), D58 (unipedal support for 10 s on the right limb), E82 (jumping on unipedal support 10 times on the right foot), and E83 (jumping on unipedal support 10 times on the left foot) were the most challenging skills, even after the intervention (Figure 2).

In dimension E, maximum improvement was observed in the following activities: perform 10 consecutive steps forward with free arms (item 69); perform 10 consecutive steps forward, stop, turn 180°, and return (item 70); kick a ball with the right foot (item 78); and climb 4 steps with unilateral support and alternating feet (item 84) (Figure 2), and an overall increase in this dimension (M = 11.58% ± SEM = 2.7% p.p., *p* = 0.002).

## 4 Discussion

In this study, we observed that the Therasuit method appeared to have a positive and significant effect on gross motor function for children with ASD. It was possible to observe that the acquisition of motor skills for maintaining and transferring to orthostasis showed greater progress compared to dynamic skills in orthostasis, gradually moving to activities that are more complex. This finding relates to motor acquisition through intervention strategies with “shaping,” as observed in constraint-induced therapy (27).

The skills primarily linked to activities such as jumping and unipedal support, vertical and frontal jumping, climbing up/down stairs without the support and with alternate feet, transferring from a semi-kneeling position to orthostasis, running (with stop and return control), and walking backward for 10 consecutive steps were observed to be more difficult for the children, even after the intervention. Such impairments are associated with deficits in cortical circuitry, possibly from alterations in the corpus callosum, hindering the connection between the cerebral hemispheres and causing delayed information processing and coordination (28).

Previous work has shown that the minimal clinically important difference (MCID) for the GMFM-88 total score is 1.1–5.3% p.p. for children with acquired brain injury and 0.1–3.0% p.p. for cerebral palsy (29). To the best of our knowledge, there is no MCID for children with ASD. However, our study has shown an improvement in the GMFM-88 total score after the Therasuit method that is greater than those observed for the MCID of children with acquired brain injury and cerebral palsy, which could indicate the clinical relevance of the method.

Our results support that the Therasuit method is a promising alternative for treating gross motor function in preschool-aged children with ASD. To the best of our knowledge, no published studies are using the Therasuit method in individuals with ASD. A previous review published in 2020 showed the Early Start Denver Model as one of the most efficient strategies for treating preschool-aged children with ASD, requiring high-intensity and frequency, multiprofessional intervention to increase learning and cortical pathways, much like the Therasuit method. Such a model supports the hypothesis of gaining motor function through the Therasuit method in children with ASD (21, 30).

However, most studies address physical therapy as traditional kinesiotherapy. Typically, motor gains are associated with increased social interaction and communication skills and other therapies and have shown to be a promising resource. This therapy promotes stimuli for verbal and nonverbal social behaviors, as well as tending to improve executive skills through motivation and encouragement to perform proposed activities (14).

The study is limited by the absence of data related to other development domains, which would allow for an interesting comparison between motor, linguistic, or cognitive aspects. Also, the study did not address the acceptability of the child and family to the Therasuit method or how this intervention impacted the child’s participation in daily life. Furthermore, the case series data cannot provide evidence for the specific effects of the Therasuit method on gross motor function in children affected by ASD. Any differential effect of the Therasuit method on improving gross motor function in children affected by ASD would have to be assessed within a control group design with children with ASD receiving either the Therasuit method or an alternative treatment. Yet, within the study design, it was possible to identify the Therasuit method as a useful resource for treating gross motor function in children with ASD.

Therasuit method increased the gross motor function of children with ASD, indicating that the method appears to be a viable resource for treating motor impairments in ASD. Further studies are needed to verify, with greater statistical strength, the effect of the Therasuit method on this population.

## Data availability statement

The raw data supporting the conclusions of this article will be made available by the authors, without undue reservation.

## Ethics statement

The studies involving humans were approved by Comitê de Ética em Pesquisa (CEP) da Universidade Federal do Amazonas (UFAM). The studies were conducted in accordance with the local legislation and institutional requirements. Written informed consent for participation in this study was provided by the participants’ legal guardians/next of kin. Written informed consent was obtained from the minor(s)’ legal guardian/next of kin for the publication of any potentially identifiable images or data included in this article.

## Author contributions

PB: Supervision, Visualization, Writing – original draft, Writing – review & editing. AF: Conceptualization, Data curation, Formal analysis, Investigation, Methodology, Resources, Writing – original draft, Writing – review & editing. TF: Conceptualization, Formal analysis, Funding acquisition, Investigation, Resources, Writing – review & editing. RF: Data curation, Writing – review & editing, Formal analysis, Visualization. CL: Data curation, Investigation, Methodology, Resources, Writing – review & editing. AM: Conceptualization, Data curation, Formal analysis, Funding acquisition, Investigation, Methodology, Project administration, Resources, Supervision, Writing – original draft, Writing – review & editing.
