# Supplementary material for: Positive impact of the Therasuit method on gross motor function of children with autism spectrum disorder: Case series
Source: Front Neurol. 2023 Dec 14;14:1254867. doi: 10.3389/fneur.2023.1254867 (PMC10760636; doi:10.3389/fneur.2023.1254867)
Supplement: Supplementary file 1 [file Data_Sheet_1.docx]

| **Check (**3**) the appropriate score:** if an item is not tested (NT), circle the item number on the right column | | | | | | | |
| --- | --- | --- | --- | --- | --- | --- | --- |
| **Item** |  | **A: LYING & ROLLING** | | **SCORE** |  |  | **NT** |
|  | 1. | SUP, HEAD IN MIDLINE: TURNS HEAD WITH EXTREMITIES SYMMETRICAL _0_□ | | _1_□ | _2_□ | _3_□ | 1. |
| ***** 2. SUP: BRINGS HANDS TO MIDLINE, FINGERS ONE WITH THE OTHER................................................... 0□ _1_□ _2_□ _3_□ 2. | | | | | | | |
|  | 3. | SUP: LIFTS HEAD 45°......................................................................................................... | _0_□ | _1_□ | _2_□ | _3_□ | 3. |
|  | 4. | SUP: FLEXES R HIP & KNEE THROUGH FULL RANGE .................................................................... | _0_□ | _1_□ | _2_□ | _3_□ | 4. |
|  | 5. | SUP: FLEXES L HIP & KNEE THROUGH FULL RANGE...................................................................... | _0_□ | _1_□ | _2_□ | _3_□ | 5. |
| ***** | 6. | SUP: REACHES OUT WITH R ARM, HAND CROSSES MIDLINE TOWARD TOY.......................................... | _0_□ | _1_□ | _2_□ | _3_□ | 6. |
| ***** | 7. | SUP: REACHES OUT WITH L ARM, HAND CROSSES MIDLINE TOWARD TOY .......................................... | _0_□ | _1_□ | _2_□ | _3_□ | 7. |
|  | 8. | SUP: ROLLS TO PR OVER R SIDE ............................................................................................ | _0_□ | _1_□ | _2_□ | _3_□ | 8. |
|  | 9. | SUP: ROLLS TO PR OVER L SIDE............................................................................................. | _0_□ | _1_□ | _2_□ | _3_□ | 9. |
| ***** 10. PR: LIFTS HEAD UPRIGHT ....................................................................................................... _0_□ _1_□ _2_□ _3_□ 10. | | | | | | | |
|  | 11. PR ON FOREARMS: LIFTS HEAD UPRIGHT, ELBOWS EXT., CHEST RAISED _0_□ | | | _1_□ | _2_□ | _3_□ | 11. |
|  | 12. PR ON FOREARMS: WEIGHT ON R FOREARM, FULLY EXTENDS OPPOSITE ARM FORWARD 0□ | | | _1_□ | _2_□ | _3_□ | 12. |
|  | 13. PR ON FOREARMS: WEIGHT ON L FOREARM, FULLY EXTENDS OPPOSITE ARM FORWARD 0□ | | | _1_□ | _2_□ | _3_□ | 13. |
|  | 14. PR: ROLLS TO SUP OVER R SIDE _0_□ | | | _1_□ | _2_□ | _3_□ | 14. |
|  | 15. PR: ROLLS TO SUP OVER L SIDE _0_□ | | | _1_□ | _2_□ | _3_□ | 15. |
|  | 16. PR: PIVOTS TO R 90° USING EXTREMITIES 0□ | | | _1_□ | _2_□ | _3_□ | 16. |
|  | 17. PR: PIVOTS TO L 90° USING EXTREMITIES 0□ | | | _1_□ | _2_□ | _3_□ | 17. |
|  | **TOTAL DIMENSION A** | | |  |  |  |  |
|  |  | | |  |  |  |  |
| **Item** | **B: SITTING** | | | **SCORE** |  |  | **NT** |
| ***** 18. SUP, HANDS GRASPED BY EXAMINER: PULLS SELF TO SITTING WITH HEAD CONTROL........... 0□ 1□ 2□ 3□ 18. | | | | | | | |
|  | 19. | SUP: ROLLS TO R SIDE, ATTAINS SITTING ................................................................................. | _0_□ | _1_□ | _2_□ | _3_□ | 19. |
|  | 20. | SUP: ROLLS TO L SIDE, ATTAINS SITTING .................................................................................. | _0_□ | _1_□ | _2_□ | _3_□ | 20. |
| ***** | 21. | SIT ON MAT, SUPPORTED AT THORAX BY THERAPIST: LIFTS HEAD UPRIGHT, MAINTAINS 3 SECONDS .......................................................................................................... | _0_□ | _1_□ | _2_□ | _3_□ | 21. |
| ***** | 22. | SIT ON MAT, SUPPORTED AT THORAX BY THERAPIST: LIFTS HEAD MIDLINE, MAINTAINS  10 SECONDS ....................................................................................................................... | _0_□ | _1_□ | _2_□ | _3_□ | 22. |
| ***** | 23. | SIT ON MAT, ARM(S) PROPPING: MAINTAINS, 5 SECONDS ................................................ | _0_□ | _1_□ | _2_□ | _3_□ | 23. |
| ***** | 24. | SIT ON MAT: MAINTAIN, ARMS FREE, 3 SECONDS .................................................................... | _0_□ | _1_□ | _2_□ | _3_□ | 24. |
| ***** | 25. | SIT ON MAT WITH SMALL TOY IN FRONT: LEANS FORWARD, TOUCHESTOY, RE-ERECTS | _0_□ | _1_□ | _2_□ | _3_□ | 25. |
| ***** | 26. | SIT ON MAT: TOUCHES TOY PLACED 45° BEHIND CHILD’S R SIDE, RETURNS TO START .................... | _0_□ | _1_□ | _2_□ | _3_□ | 26. |
| ***** | 27. | SIT ON MAT: TOUCHES TOY PLACED 45° BEHIND CHILD’S L SIDE, RETURNS TO START..................... | _0_□ | _1_□ | _2_□ | _3_□ | 27. |
|  | 28. | R SIDE SIT: MAINTAINS, ARMS FREE, 5 SECONDS ..................................................................... | _0_□ | _1_□ | _2_□ | _3_□ | 28. |
|  | 29. | L SIDE SIT: MAINTAINS, ARMS FREE, 5 SECONDS ..................................................................... | _0_□ | _1_□ | _2_□ | _3_□ | 29. |
| ***** | 30. | SIT ON MAT: LOWERS TO PR WITH CONTROL .......................................................................... | _0_□ | _1_□ | _2_□ | _3_□ | 30. |
| ***** | 31. | SIT ON MAT WITH FEET IN FRONT: ATTAINS 4 POINT OVER R SIDE ................................... | _0_□ | _1_□ | _2_□ | _3_□ | 31. |
| ***** | 32. | SIT ON MAT WITH FEET IN FRONT: ATTAINS 4 POINT OVER L SIDE .................................... | _0_□ | _1_□ | _2_□ | _3_□ | 32. |
|  | 33. | SIT ON MAT: PIVOTS 90°, WITHOUT ARMS ASSISTING .............................................................. | _0_□ | _1_□ | _2_□ | _3_□ | 33. |
| ***** | 34. | SIT ON BENCH: MAINTAINS, ARMS AND FEET FREE, 10 SECONDS............................................... | _0_□ | _1_□ | _2_□ | _3_□ | 34. |
| ***** | 35. | STD: ATTAINS SIT ON SMALL BENCH ......................................................................................... | _0_□ | _1_□ | _2_□ | _3_□ | 35. |
| ***** | 36. | ON THE FLOOR: ATTAINS SIT ON SMALL BENCH ..................................................................... | _0_□ | _1_□ | _2_□ | _3_□ | 36. |
| ***** | 37. | ON THE FLOOR: ATTAINS SIT ON LARGE BENCH ..................................................................... | _0_□ | _1_□ | _2_□ | _3_□ | 37. |
|  |  |  |  |  |  |  |  |
|  |  | **TOTAL DIMENSION B** |  |  |  |  |  |

WITHOUT ARM PROPPING ........................................................................................................

| **Item** | **C: CRAWLING & KNEELING** |  | **SCORE** | **NT** |
| --- | --- | --- | --- | --- |
| 38. | PR: CREEPS FORWARD 1.8m (6') .......................................................................................... | _0_□ | _1_□ _2_□ _3_□ | 38. |
| ***** 39. | 4 POINT: MAINTAINS, WEIGHT ON HANDS AND KNEES, 10 SECONDS ............................................... | _0_□ | _1_□ _2_□ _3_□ | 39. |
| ***** 40. | 4 POINT: ATTAINS SIT ARMS FREE .......................................................................................... | _0_□ | _1_□ _2_□ _3_□ | 40. |
| ***** 41. | PR: ATTAINS 4 POINT, WEIGHT ON HANDS AND KNEES .................................................................. | _0_□ | _1_□ _2_□ _3_□ | 41. |
| ***** 42. | 4 POINT: REACHES FORWARD WITH R ARM, HAND ABOVE SHOULDER LEVEL .................................... | _0_□ | _1_□ _2_□ _3_□ | 42. |
| ***** 43. | 4 POINT: REACHES FORWARD WITH L ARM, HAND ABOVE SHOULDER LEVEL..................................... | _0_□ | _1_□ _2_□ _3_□ | 43. |
| ***** 44. | 4 POINT: CRAWLS OR HITCHES FORWARD 1.8m(6').................................................................. | _0_□ | _1_□ _2_□ _3_□ | 44. |
| ***** 45. | 4 POINT: CRAWLS RECIPROCALLY FORWARD1.8m ( 6')............................................................. | _0_□ | _1_□ _2_□ _3_□ | 45. |
| ***** 46. | 4 POINT: CRAWLS UP 4 STEPS ON HANDS AND KNEES/FEET ......................................................... | _0_□ | _1_□ _2_□ _3_□ | 46. |
| 47. | 4 POINT: CRAWLS BACKWARDS DOWN 4 STEPS ON HANDS AND KNEES/FEET ................................... | _0_□ | _1_□ _2_□ _3_□ | 47. |
| ***** 48. | SIT ON MAT: ATTAINS HIGH KN USING ARMS, MAINTAINS, ARMS FREE, 10 SECONDS ......................... | _0_□ | _1_□ _2_□ _3_□ | 48. |
| 49. | HIGH KN: ATTAINS HALF KN ON R KNEE USING ARMS, MAINTAINS, ARMS FREE, 10 SECONDS .............. | _0_□ | _1_□ _2_□ _3_□ | 49. |
| 50. | HIGH KN: ATTAINS HALF KN ON L KNEE USING ARMS, MAINTAINS, ARMS FREE, 10 SECONDS ............... | _0_□ | _1_□ _2_□ _3_□ | 50. |
| ***** 51. | HIGH KN: KN WALKS FORWARD 10 STEPS, ARMS FREE .............................................................. | _0_□ | _1_□ _2_□ _3_□ | 51. |
|  |  |  |  |  |
|  | **TOTAL DIMENSION C** |  |  |  |

| **Item** | **D: STANDING** |  | **SCORE** | **NT** |
| --- | --- | --- | --- | --- |
| ***** 52. | ON THE FLOOR: PULLS TO STD AT LARGE BENCH ................................................................... | _0_□ | _1_□ _2_□ _3_□ | 52. |
| ***** 53. | STD: MAINTAINS, ARMS FREE, 3 SECONDS ................................................................................ | _0_□ | _1_□ _2_□ _3_□ | 53. |
| ***** 54. | STD: HOLDING ON TO LARGE BENCH WITH ONE HAND, LIFTS R FOOT, 3 SECONDS .............................. | _0_□ | _1_□ _2_□ _3_□ | 54. |
| ***** 55. | STD: HOLDING ON TO LARGE BENCH WITH ONE HAND, LIFTS L FOOT, 3 SECONDS ............................... | _0_□ | _1_□ _2_□ _3_□ | 55. |
| ***** 56. | STD: MAINTAINS, ARMS FREE, 20 SECONDS .............................................................................. | _0_□ | _1_□ _2_□ _3_□ | 56. |
| ***** 57. | STD: LIFTS L FOOT, ARMS FREE, 10 SECONDS .......................................................................... | _0_□ | _1_□ _2_□ _3_□ | 57. |
| ***** 58. | STD: LIFTS R FOOT, ARMS FREE, 10 SECONDS ......................................................................... | _0_□ | _1_□ _2_□ _3_□ | 58. |
| ***** 59. | SIT ON SMALL BENCH: ATTAINS STD WITHOUT USING ARMS................................................... | _0_□ | _1_□ _2_□ _3_□ | 59. |
| ***** 60. | HIGH KN: ATTAINS STD THROUGH HALF KN ON R KNEE, WITHOUT USING ARMS................................. | _0_□ | _1_□ _2_□ _3_□ | 60. |
| ***** 61. | HIGH KN: ATTAINS STD THROUGH HALF KN ON L KNEE, WITHOUT USING ARMS ................................. | _0_□ | _1_□ _2_□ _3_□ | 61. |
| ***** 62. | STD: LOWERS TO SIT ON FLOOR WITH CONTROL, ARMS FREE ......................................................... | _0_□ | _1_□ _2_□ _3_□ | 62. |
| ***** 63. | STD: ATTAINS SQUAT, ARMS FREE ........................................................................................... | _0_□ | _1_□ _2_□ _3_□ | 63. |
| ***** 64. | STD: PICKS UP OBJECT FROM FLOOR, ARMS FREE, RETURNS TO STAND ............................................ | _0_□ | _1_□ _2_□ _3_□ | 64. |
|  |  |  |  |  |
|  | **TOTAL DIMENSION D** |  |  |  |

| **Item** | **E: WALKING, RUNNING & JUMPING** |  | **SCORE** | **NT** |
| --- | --- | --- | --- | --- |
| ***** 65. | STD, 2 HANDS ON LARGE BENCH: CRUISES 5 STEPS TO R ............................... | _0_□ | _1_□ _2_□ | 3□ 65. |
| ***** 66. | STD, 2 HANDS ON LARGE BENCH: CRUISES 5 STEPS TO L ................................ | _0_□ | _1_□ _2_□ | 3□ 66. |
| ***** 67. | STD, 2 HANDS HELD: WALKS FORWARD 10 STEPS ................................................ | _0_□ | _1_□ _2_□ | 3□ 67. |
| ***** 68. | STD, 1 HAND HELD: WALKS FORWARD 10 STEPS................................................... | _0_□ | _1_□ _2_□ | 3□ 68. |
| ***** 69. | STD: WALKS FORWARD 10 STEPS ............................................................................ | _0_□ | _1_□ _2_□ | 3□ 69. |
| ***** 70. | STD: WALKS FORWARD 10 STEPS, STOPS, TURNS 180°, RETURNS ................................. | _0_□ | _1_□ _2_□ | 3□ 70. |
| ***** 71. | STD: WALKS BACKWARD 10 STEPS ........................................................................... | _0_□ | _1_□ _2_□ | 3□ 71. |
| ***** 72. | STD: WALKS FORWARD 10 STEPS, CARRYING A LARGE OBJECT WITH 2 HANDS.................... | _0_□ | _1_□ _2_□ | 3□ 72. |
| ***** 73. | STD: WALKS FORWARD 10 CONSECUTIVE STEPS BETWEEN PARALLEL LINES 20cm (8")APART | _0_□ | _1_□ _2_□ | 3□ 73. |
| ***** 74. | STD: WALKS FORWARD 10 CONSECUTIVE STEPS ON A STRAIGHT LINE 2cm (3/4") WIDE ........... | _0_□ | _1_□ _2_□ | 3□ 74. |
| ***** 75. | STD: STEPS OVER STICK AT KNEE LEVEL, R FOOT LEADING ............................................. | _0_□ | _1_□ _2_□ | 3□ 75. |
| ***** 76. | STD: STEPS OVER STICK AT KNEE LEVEL, L FOOT LEADING ............................................. | _0_□ | _1_□ _2_□ | 3□ 76. |
| ***** 77. | STD: RUNS 4.5m (15’), STOPS & RETURNS................................................................ | _0_□ | _1_□ _2_□ | 3□ 77. |
| ***** 78. | STD: KICKS BALL WITH R FOOT ................................................................................ | _0_□ | _1_□ _2_□ | 3□ 78. |
| ***** 79. | STD: KICKS BALL WITH L FOOT................................................................................. | _0_□ | _1_□ _2_□ | 3□ 79. |
| ***** 80. | STD: JUMPS 30cm (12") HIGH, BOTH FEET SIMULTANEOUSLY ........................................ | _0_□ | _1_□ _2_□ | 3□ 80. |
| ***** 81. | STD: JUMPS FORWARD 30 cm (12"), BOTH FEET SIMULTANEOUSLY ................................ | _0_□ | _1_□ _2_□ | 3□ 81. |
| ***** 82. | STD ON R FOOT: HOPS ON R FOOT 10 TIMES WITHIN A 60cm (24") CIRCLE............... | _0_□ | _1_□ _2_□ | 3□ 82. |
| ***** 83. | STD ON L FOOT: HOPS ON L FOOT 10 TIMES WITHIN A 60cm (24") CIRCLE ................ | _0_□ | _1_□ _2_□ | 3□ 83. |
| ***** 84. | STD, HOLDING 1 RAIL: WALKS UP 4 STEPS, HOLDING 1 RAIL, ALTERNATING FEET ......... | _0_□ | _1_□ _2_□ | 3□ 84. |
| ***** 85. | STD, HOLDING 1 RAIL: WALKS DOWN 4 STEPS, HOLDING 1 RAIL, ALTERNATING FEET .... | _0_□ | _1_□ _2_□ | 3□ 85. |
| ***** 86. | STD: WALKS UP 4 STEPS, ALTERNATING FEET .............................................................. | _0_□ | _1_□ _2_□ | 3□ 86. |
| ***** 87. | STD: WALKS DOWN 4 STEPS, ALTERNATING FEET ......................................................... | _0_□ | _1_□ _2_□ | 3□ 87. |
| ***** 88. | STD ON 15cm (6") STEP: JUMPS OFF, BOTH FEET SIMULTANEOUSLY .......................... | _0_□ | _1_□ _2_□ | 3□ 88. |
|  | **TOTAL DIMENSION E** |  |  |  |

## Was this assessment indicative of this child’s “regular” performance? YES 🞏 NO 🞏

COMMENTS:

# GMFM-88 SUMMARY SCORE

### DIMENSION CALCULATION OF DIMENSION % SCORES

**GOAL AREA**

(indicated with ✓ check)

1. Lying & Rolling Total Dimension A = × 100 = % A. 🞏

51 51

## Sitting Total Dimension B = × 100 = % B. 🞏

60 60

## Crawling & Kneeling Total Dimension C = × 100 = % C. 🞏

42 42

## Standing Total Dimension D = × 100 = % D. 🞏

39 39

## Walking, Running & Jumping

Total Dimension E = × 100 = % E. 🞏

## 72 72

**TOTAL SCORE =** %A + %B + %C + %D + %E

## Total # of Dimensions

**=** = = % 5

**GOAL TOTAL SCORE =** Sum of %scores for each dimension identified as a goal area

## # of Goal areas

**=** = %

GMFM-66 Gross Motor Ability Estimator Score 1

GMFM-66 Score =

previous GMFM-66 Score =

to

95% Confidence Intervals

to

95% Confidence Intervals

change in GMFM-66 =

1 from the Gross Motor Ability Estimator (GMAE-2) Software
